# Promoting physical activity: development and testing of self-determination theory-based interventions

**DOI:** 10.1186/1479-5868-9-20

**Published:** 2012-03-02

**Authors:** Michelle S Fortier, Joan L Duda, Eva Guerin, Pedro J Teixeira

**Affiliations:** 1 School of Human Kinetics, University of Ottawa, Ottawa K1N 6N5, Canada; 2 School of Sport and Exercise Sciences, University of Birmingham, Birmingham B15 2TT, UK; 3 Faculty of Human Kinetics, Technical University of Lisbon, 1495-710 Cruz Quebrada, Portugal

## Abstract

A growing number of studies have pulled from Deci and Ryan's Self-Determination Theory to design interventions targeting health behavior change. More recently, researchers have begun using SDT to promote the adoption and maintenance of an active lifestyle. In this review, we aim to highlight how researchers and practitioners can draw from the SDT framework to develop, implement, and evaluate intervention efforts centered on increasing physical activity levels in different contexts and different populations. In the present paper, the rationale for using SDT to foster physical activity engagement is briefly reviewed before particular attention is given to three recent randomized controlled trials, the Canadian Physical Activity Counseling (PAC) Trial, the Empower trial from the UK, and the Portuguese PESO (Promotion of Health and Exercise in Obesity) trial, each of which focused on promoting physical activity behavior. The SDT-based intervention components, procedures, and participants are highlighted, and the key findings that have emanated from these three trials are presented. Lastly, we outline some of the limitations of the work conducted to date in this area and we acknowledge the challenges that arise when attempting to design, deliver, and test SDT-grounded interventions in the context of physical activity promotion.

## Introduction

In this paper, we build a case for Self-Determination Theory's (SDT) [1,2] relevance in the design, delivery and testing of physical activity (PA) interventions. To support our arguments, we begin by briefly highlighting the growing literature grounded in this theoretical framework within the physical activity context. Then, and as the central focus of this paper, we review in detail three large scale randomized controlled trials that have formally tested SDT in applied settings to promote increases in PA, namely (a) the Physical Activity Counseling (PAC) trial conducted in a primary health care setting and based in Canada [3,4], (b) the "Empower" trial, a randomized controlled trial within an exercise on referral scheme in the United Kingdom [5,6] and (c) the "PESO" (Promotion of Health and Exercise in Obesity) trial, conducted in a community context in Portugal [7,8]. We will briefly summarize the main findings from these studies, describe common lessons derived from them, and discuss current challenges and limitations faced in this line of work, including those ensuing from balancing efficacy with effectiveness in real-world intervention research. We will close with suggested avenues for future SDT-grounded intervention research in PA promotion.

## Self-Determination Theory and Physical Activity

Self-Determination Theory (SDT) [1,2] is a motivational theory that has received significant research attention and support in predicting PA as well as in the development of PA interventions. SDT draws a distinction between intrinsic motivation, which involves engaging in a behavior for its own sake (i.e., for challenge and enjoyment), and extrinsic forms of motivation. The latter involves doing an activity because it is instrumental to achieving a separate consequence and this can be experienced as heteronomous (i.e., controlling) or autonomous to varying degrees. SDT proposes a continuum for the internalization of motivation, whereby individuals become more autonomous (or self-determined) to engage in behaviours over time as their extrinsic motives or reasons become more internalized. Facilitation of this internalization process has been found to nurture more autonomous motivation with an ensuing predictive influence on adaptive outcomes such as behavioural engagement/persistence and well-being [9,10].

According to SDT, it is those social environments that support individuals' basic psychological needs specifically (i.e., autonomy, relatedness, and competence) that are assumed to foster more autonomous motivational patterns as well as adaptive outcomes. When individuals are more autonomously motivated, otherwise referred to as 'being self-determined', "they experience volition, or a self-endorsement of their actions" [10]. The highest level of self-determination is intrinsic motivation where behaviours, such as PA, are performed for their own inherent rewards, such as enjoyment or challenge. Readers are encouraged to consult Deci and Ryan [9] and also Patrick & Williams [11] in this issue for summaries of the basic theoretical premises of SDT, including descriptions of the different motivational regulations along the continuum. Similarities and differences between SDT and other theoretical/counselling approaches can also be found elsewhere (e.g., Motivational Interviewing; [11,12]).

SDT has received significant empirical support in the context of health behavior change [13,14] and in the physical activity context specifically [15-17]. One of the strengths of SDT is that it offers malleable processes of behavioral change that can be targeted in different health behaviour interventions [4]. Essentially, SDT researchers can develop and implement intervention strategies that are purported to satisfy the three basic psychological needs, thus fostering internalization and positive behavior change, in this case, adoption and maintenance of PA. Broadly, the purpose of SDT interventions is to assist individuals' progress on the continuum towards more autonomous forms of motivation. Unlike with other health behaviours (e.g., brushing teeth, wearing a seat belt) which are less intrinsically satisfying, intrinsic motivation can be targeted to a considerable extent in the case of PA by honing in on people's natural interest and enjoyment in activities such as sports, dancing, water activities, etc. [13,18].

**Figure 1:**
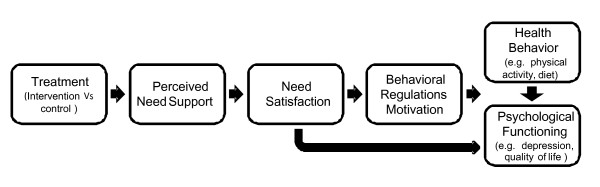
**The SDT process model for health behavior change in intervention research**.

Overall it is said that when the motivational determinants and consequences of the complete SDT causal sequence (Figure 1, description to follow) are fused together, this creates the outline of interventions that can be quite powerful [19]. Such intervention developments have been achieved successfully in many lines of health care research, for example, diabetes control, smoking cessation, diet, weight loss, etc. [11,20-22]. In fact, given the solid support for SDT's principles in the PA realm, and the evidence that providing a need-supportive context can lead to successful health behavior change, researchers have begun to implement and evaluate PA promotion interventions grounded in SDT.

In an early study, Levy and Cardinal [23] examined the influence of an SDT-based PA intervention on women' PA behavior using a mail-mediated approach (i.e., packets with strategies designed to promote the three needs) and contrasted this with a control condition. Although the women in this RCT showed improvements in PA and autonomous motivation, this occurred irrespective of treatment. These results unfortunately provided little support for this mail-delivered attempt to promote participants' psychological needs. This was perhaps due to low participant compliance or the low intensity of the intervention rather than any shortcomings in the application value of SDT principles. Notwithstanding these findings, recent attention has turned to technologically innovative means of delivering SDT-inspired information regarding PA. Specifically, researchers are testing SDT-based computerized interventions, based on personal digital assistants (PDAs) or the Internet, to improve participation in PA [24,25]. Although final results are not yet available, feasibility data and preliminary evidence of intervention impact on targeted motivational processes appear promising.

Edmunds, Ntoumanis, and Duda [26] tested the impact of an SDT-inspired teaching style on need satisfaction, PA motivation regulations, and related PA behavior in female exercise class participants. They compared participants exposed to a teaching style that was "autonomy supportive, well-structured, and interpersonally involving" (p. 375) to those receiving the typical teaching style observed in this context. Overall, their results supported the premises of SDT in that those participants exposed to the former condition demonstrated greater need satisfaction and this predicted being more autonomously motivated to engage in PA. Moreover, PA behavior (i.e., class attendance) was significantly greater for those participating in a class marked by an SDT-inspired teaching style.

Recently, three longer-term and large-scaled SDT-based randomized controlled trials were conducted which focused on PA behavior change. In each case, emphasis was placed on implementing strategies that should facilitate the satisfaction of the three psychological needs. They focused heavily on promoting both autonomous motivation for PA engagement as well as PA behavior itself. Although the trials took place in distinct contexts, all trials sought subsequent improvements in health outcomes in 'at risk' study participants. A common goal of these trials was to test some or all of the sequential steps of the motivational process model proposed by SDT, as depicted in Figure 1. This model generally depicts the sequential causal mechanisms thought to precede lasting behaviour change (and beneficial psychological well-being) with health interventions as the starting point. More details and an empirical test of this general sequence is available elsewhere [7,27].

While varied in duration and study protocol, these more 'intensive' interventions shared many of the same principles and were all implemented in a predominantly face-to-face format (comparisons of background characteristics and elements of delivery can be found in Table 1 and Table 2, and methodological characteristics related to assessment in Table 3). The principles which undergirded the three trials, along with noteworthy findings, are described in detail in the sections that follow.

**Table 1:** Background characteristics of three key SDT-based intervention trials for PA promotion.

Descriptor	PAC Trial	Empower Trial	PESO Trial
Trial location	Canada	UK	Portugal
Participant gender	69% female; 31% male.	72.9% female; 27.1% male.	100% female.
Participant age & education	*M*_age _= 47.3; years of education *M *= 14.75.	*M*_age _= 49.3; Education = NA	*M*_age _= 38.1; 66% with higher education.^a^
Participant baseline PA & weight status	< 150 min of mod PA per week; *M*_BMI _= 30.74.	< 150 min of mod PA per week; M_BMI _= 33.21^a^	*M *_min/week _of PA = 110.2; M_BMI _= 31.7.^a^
Intervention setting	Inactive *patients *in a large community-based primary care practice (total of 5 HCP).	Primary care *patients *referred (by HCP) to Exercise on Prescription (EoP) scheme at community leisure centres.	*Participants *recruited via local advertisement (e.g., schools, health care centers) for a weight management program at the Technical University of Lisbon.
Domain	PA	PA	Weight control (emphasis on PA & nutrition).

Abbreviations: PA = physical activity; HCP = Health care provider(s); EoP = Exercise on Prescription

^a^Intervention group stats only. No significant differences found with control group.

**Table 2:** Methodological characteristics (delivery) of three key SDT-based intervention trials for PA promotion.

Descriptor	PAC Trial	Empower Trial	PESO Trial
Randomization	- Participants randomized to 2 groups (statistician-created sequence): BPAC = brief or IPAC = intensive). - Stratified by gender & age.	- 13 leisure centres with EoP randomized to 2 arms (statistician-created sequence): standard practice (control) or SDT-based intervention - Stratified by Primary Care Trust (PCT) and deprivation of population served	Participants from 3 annual cohorts randomized (Excel 2007 random number generator function) to 2 groups: experimental (SDT-based intervention) or control.
Interventionist(s)	- BPAC (control) by HCP. - IPAC by PA counsellor (part of health care team; exercise science degree).	- Control/intervention: HFAs at community leisure centres. - HFAs: Level 3 National Occupational Standards.	Intervention (staff): 6 Ph.D. or M.S. level exercise physiologists, nutritionists, dieticians, & psychologists. Control: invited lecturers
Interventionist(s) training	- BPAC (HCP): 3 training seminars (SDT-based delivery of first 4A's of 7A's model; [3]) - IPAC (PA counsellor): 2-month autonomy-support & MI training (last 3 A's) + counselling skills + bi-weekly supervision.	- Control HFAs: Training Day One (see [11]) + 2^nd ^training meeting (on topic of recruitment). - Intervention HFAs: Training Day One & Two + 1/2 day (theory, client-centered communication, creating autonomy-supportive climate).	- Intervention staff: 2-day-long workshop and 4-6 training meetings provided by in-house SDT experts and by one external MI expert. - Control: no specific weight control or behavior change/SDT training
Intervention duration/ intensity	- IPAC: BPAC + intensive 3-month PA-counselling (last 3 A's; 6 sessions, every 2 weeks). - IPAC sessions: 3 in-person, 3 conducted by phone; see [3] for format/order of sessions).	- Control/intervention: initial 1 hour consultation + 3-month program (6-month follow-up). - One-on-one contacts (3) each month; telephone or in-person; intended: 5 to 30 min per contact.	- Consistent across groups. - One-year intervention (group-based); 2-year follow-up. - Thirty (29 for control) weekly or bi-weekly sessions of 120-min each.
Control group	BPAC: 2-4 minute PA intervention delivered by HCP (first 4 A's of 7A's + PA prescription).	Standard practice (control) of each EoP leisure centre provided by HFA *without *SDT training.	General health education curriculum (themed courses including stress management, food safety and preventive nutrition, interpersonal relationships, etc.).

Abbreviations: BPAC = Brief physical activity counselling; IPAC: Intensive physical activity counselling, PA = physical activity; EoP = Exercise on prescription; SDT = Self-determination theory; HCP = Health care provider(s); HFA = health and fitness advisor; MI = Motivational interviewing

**Table 3:** Methodological characteristics (assessment) of three key SDT-based intervention trials for PA promotion.

Descriptor	PAC Trial	Empower Trial	PESO Trial
Main SDT components	- The PAC 7A's model of behavioural counseling steps [32] shared between HCP & PA counselor (*autonomy-supportive*) - First 4 A's (BPAC): Address, Ask, Advice, & Assess/Agree (with PA prescription). - Last 3 A's (IPAC): Assess, Assist, and Arrange. - IPAC: values interview ^b^, PA goals (discuss, support, encourage), problem solving (barriers), enjoyment enhancement, discussing relapse & suggestions for maintenance.	- Autonomy supportive protocol for health counsellors [36]. - Individualized discussions: risk/benefits, integration of PA with life values^b^, perceptions of barriers/resources for change. - HFA: listening, parroting/paraphrasing, handling resistance, double sided reflection. ^b^ - Failure normalization & recalibration of implementation plans (HFA & patient together). - Targeting feelings during PA. - HFA support of patient internalisation of PA involvement.	- Promoting sense of ownership & an internal locus of causality: choice & self-initiation, congruence of values, life goals, lifestyles.^b^ - Structure: safety, goals, PA monitoring, barriers, positive feedback (informational). - Provision of options and decisions for PA; encouraging enjoyment, self-initiation, & independent problem solving. -Exploration of participants' own motivations & goals.
Main SDT outcomes	(i) *Quantity *of motivation; (ii) perceived competence; (iii) autonomous motivation; (iv) PA motivation/regulations; (v) perceptions of autonomy support (climate)	(i) PA motivation/regulations; (ii) perceptions of PA-based needs satisfaction (competence, autonomy, relatedness); (iii) perceptions of autonomy support (climate);	(i) Perceptions of need-support (climate); (ii) treatment & exercise autonomous regulations; (iii) PA participation motives; (iv) locus of causality; (v) perceived competence & enjoyment for PA.
Main SDT-based measures	(i) graded approach;^a ^(ii) per- ceived competence in exercise scale [45]; (iii) TSRQ [45]; (iv) BREQ-2 [44]; (v) HCCQ [45].	(i) BREQ-2 [44]; (ii) Psy-chological Needs in Exercise Scale [55]; (iii) HCCQ [45].	(i) HCCQ [45]; (ii) Treatment and Exercise Self-Regulation Questionnaire [64,65]; (iii) EMI-2 [66]; (iv) the Exercise Locus of Causality scale [67]; (v) Intrinsic Motivation Inventory [68].
Assessment time points	BPAC & IPAC: Baseline, 6-, 13-, 19-, & 25-weeks	Control/intervention: Baseline, 3- & 6-months	Control/intervention: baseline, 4- months, 1-, 2-, & 3-year

Abbreviations: SDT = Self-determination theory; BPAC = Brief physical activity counselling; IPAC = Intensive physical activity counselling; HCP = Health care provider(s); PA = physical activity; HFA = health and fitness advisor; TSRQ = Treatment Self-Regulation Questionnaire; BREQ-2 = Behavioral Regulations in Exercise Questionnaire-2; HCCQ = Health Care Climate Questionnaire; EMI-2 = Exercise Motivation Inventory-2

^a^See PAC section for description

^b^Elements borrowed from motivational interviewing (MI)

## The Physical Activity Counseling (PAC) Trial

Studies have shown that physicians adopting autonomy-supportive interaction styles can positively influence patients' autonomous motivation and subsequent health behavior change [14,28,29]. Since family physicians are important resources for promoting PA due to their high reach and credibility, Fortier, Hogg, and colleagues [3] argued that one key way to increase population PA levels is through autonomy supportive PA counseling in primary care. However, this context poses unique challenges, such as physicians' time constraints and the modest and short-lived effect of physician counseling on patients' PA levels [30,31]. Therefore, it was proposed in the Physical Activity Counseling (PAC) trial that a specialized professional, that is, a physical activity counselor,^1 ^be integrated as a member of the primary health care team to promote PA [3]. Hence, the objective of this trial was to assess the influence of integrating a physical activity counselor trained to deliver autonomy supportive intensive counseling on participants' PA motivation and their PA levels. There were 2 arms: 1) brief autonomy supportive counseling (by the family physician) and 2) intensive counseling (brief counseling plus intensive autonomy supportive counseling by the PA counselor).

The PAC 7As model [32] was developed to guide the intervention within this primary-care-based PA promotion effort. The seven behavioral counseling steps (As) were based mainly on the As public health approach [33,34]. The As approach was originally developed to help organize clinicians' endeavours toward facilitating behavioural counselling with their patients [34]. Both the U.S. Public Health Service and the Canadian Task Force on Preventive Health Care advocate for the As constructs to be used across behavioural domains [34] while other experts have specifically advocated using the As in PA counselling [35]. There are several tasks that exist within each A and these can be accomplished through a number of modalities from print materials to in-person discussions [35].

What is innovative about the PAC 7 As model is that the steps (As) are shared amongst the primary care provider and the PA counselor to provide a shared-care collaborative approach and that these steps were to be delivered in an autonomy supportive manner. The first four As of the model (i.e., Address, Ask, Advise, & Assess/Agree), including a written PA prescription, were delivered to all participants in a 2 to 4 minute (brief) autonomy supportive counseling intervention by the family physician (BPAC). The remaining As (Assess, Assist, and Arrange) were supplied by the PA counselor to the second arm of participants in a 3-month (6 bi-weekly sessions) intensive autonomy supportive counseling intervention (IPAC). There were three in-person sessions each lasting 40-minutes, with the exception of the 60-minute initial session, as well as three 20-minute telephone sessions. The purpose, content and format of IPAC sessions are summarized elsewhere [see [3]]. IPAC was based on an SDT intervention model of PA behavior change [see [3,13]] inspired by Williams and colleagues' process model [36] and by Markland et al's integrated SDT- MI (Motivational interviewing) model [37].

The goal of IPAC was to first develop quantity of motivation then to hone quality (autonomous) motivation. *Quantity of motivation *is best represented as a gradation of the strength, amount or degree of motivation towards a specific task [38]. The concept of intentions from the Theory of Planned Behavior [39,40] aligns well with the quantity of motivation conceptualization and has been found to be consistently linked with PA behavior [41]. In the PAC trial quantity of motivation was measured using a graded approach recommended by Bandura [38] whereby participants indicated, from 0-100%, their degree of motivation to increase PA from 1 to 7 days a week.

The PAC intervention model was based on three key features: (1) it sequenced and prioritized the psychological needs within SDT (i.e., relatedness and autonomy to be developed first); (2) it detailed the intervention components (including some motivational interviewing techniques (MI) [42] according to the needs that they facilitate, similar to Markland and colleagues, and (3) it supplied an enjoyment enhancement component. The active ingredients of the intervention were: 1) a values interview, 2) discussing PA goals, 3) providing support and encouragement toward these goals, 4) problem solving barriers, 5) discussing enjoyment enhancement, 6) discussing relapse and suggestions to maintain PA all within an autonomy supportive climate.

To test the intervention, a 2-arm stratified randomized controlled design using a mixed-methods approach was used. The participants were patients from a large and diverse primary care practice in Ottawa, Canada, that were randomly assigned to either the brief counseling condition (i.e., BPAC; n = 59) or the intensive counseling condition (i.e., BPAC and IPAC; n = 61). The overall sample (N = 120) ranged in age from 20 to 67 years (*M *= 47.3), held a moderately high level of education (14.75 years), was predominantly female (69%), Caucasian (96.7%), and francophone (88.3%), and had a high BMI (30.74). Participants reported less than 150 min/week of PA [Godin Leisure Time Exercise Questionnaire mean score = 18.14] and held no uncontrolled medical condition.

Participants in the PAC trial responded to several assessments throughout the trial in order to evaluate the outcomes of interest (i.e., motivational mediators, PA, health outcomes: see Table 3 for complete details). Notably, participants were administered packages of questionnaires previously demonstrated to have psychometrically sound

characteristics in order to measure the noted outcomes at several time-points during the trial (i.e., baseline, 6-weeks [midpoint], 13-weeks [endpoint]) and post-intervention (i.e., 19- and 25- weeks). The Godin Leisure Time Exercise Questionnaire (LTEQ) [43] was employed to assess PA, whereby the number of sessions per week of mild, moderate, and strenuous activity were multiplied by corresponding intensity factors (3, 5, and 9 respectively) to yield a total score. Key SDT-measures included the Behavioral Regulations in Exercise Questionnaire-2 (BREQ-2) [44], the Health Care Climate Questionnaire (HCCQ) [45], and the Perceived Competence in Exercise Scale [45] to assess PA motivation, autonomy support, and perceived competence respectively. All participants completed quality of life measures at baseline, 13 weeks, and 25 weeks, and one third of all participants were also randomly assigned to physical and metabolic testing (e.g. VO_2 _max test) at these same 3 time-points.

The analyses and results of the PAC trial to date have revealed a number of interesting findings. Firstly, there was a medium sized overall effect (partial η^2 ^= 0.066) of counseling group on *quantity *of motivation at each time-point including follow-up; as expected, levels were significantly higher in the intensive counseling group [46]. This suggests that intensive motivational counseling can be effective at building quantity of motivation in participants and that these motivational effects are sustainable after the intervention. In addition, results showed that the gains in quantity of motivation *during *the intervention translated into increases in PA during the intervention. Specifically, there was an adjusted difference of 7.6 and 8 units on the LTEQ between the two groups at 6 weeks (*F_(1,116) _*= 7.07, p = 0.009) and 13 weeks (*F_(1,116) _*= 6.92, p = 0.010), respectively [47]. This is equivalent to two extra sessions of 20 minutes of PA per week, with one being of moderate activity (1 × 5 units of intensity) and another of mild activity (1 × 3 units of intensity). Subsequent body composition improvements *post*-intervention (e.g., effect size [ES] of 0.89 for percent body fat) were also found [47].

In keeping with the PAC trial's grounding in the SDT framework, several findings regarding the intervention's influence on *quality *of motivation (i.e., autonomous motivation) deserve mention. Specifically, after six weeks (two counseling sessions), participants in the intensive counseling condition showed significantly higher autonomous motivation scores than participants in the brief counseling condition [F(1, 117) = 4.47, p < .05, partial η^2 ^= .04] [4]. Moreover, results of a path analysis generally supported the Williams et al. [36] model in that for the intensive counseling condition, 6-week autonomous motivation and 6-week perceived competence respectively were directly and positively related to 13-week PA [4].

Lastly, it was deemed insightful to examine the interplay between quantity and quality of motivation in predicting effects of the PAC intervention via a moderated mediation [48]. Such analyses were warranted given the independent influence of quantity and quality of motivation on participants' PA in this trial and the limited existing research examining their interaction. Results revealed firstly, as predicted, that quantity of motivation mediated the intervention-PA relationship, and secondly that there was a significant interaction (β = .18, p < .05) between quantity and quality of motivation in predicting PA levels [48]. Specifically, moderate to high *autonomous *motivation was found to *increase *the effect of motivation quantity on PA.

In sum, the findings from the PAC trial suggest that SDT-MI trained PA counselors can, by fostering both quantity *and *quality of motivation, provide a valuable contribution in facilitating changes in PA behavior.

## The Empower Trial: A Self-Determination Promotive Exercise on Referral Program

As previously mentioned, growing emphasis is being placed on the role of the primary care setting in the promotion of an active lifestyle. With the long-term aims of reducing disease and promoting health in the larger community, the National Health Service in the UK has developed exercise on referral schemes to foster PA adoption and maintenance in sedentary patients [49,50]. Within these schemes, the doctor/general practitioner or practice nurse can refer a patient with at least one major risk factor for cardiovascular disease to a health and fitness advisor (HFA) at a community leisure centre. The purpose of the Empower trial was to compare the effect (at 3 and 6 months) of a PA promotion program delivered by an HFA trained in SDT principles and strategies with the standard provision exercise on referral scheme within a large British city (full details regarding the protocol are provided in Jolly et al.) [6].

The 13 leisure centres offering exercise on referral in the targeted community were randomised to the intervention or standard provision arm, and all participants in the referral scheme had the intervention consistent with the HFA at their particular leisure centre. The primary outcome in this trial was self-reported PA using the 7-Day Physical Activity Recall [51]. Perceived physical health outcomes, mental and emotional well being, and quality of life were also assessed using the Dartmouth COOP Charts [52], Subjective Vitality Scale [53] and Hospital Anxiety Depression Scale [54]. Motivation-related processes of change were examined via the BREQ-2 [44] and the Psychological Needs in Exercise Scale [55]. At the conclusion of the initial 1 hour consultation and again at the end of the 3 month program, the HCCQ [45] was employed to determine patients' perceptions of the degree of autonomy support provided by their HFA.

Within the 1-hour initial consultation within the standard exercise on referral provision, the scheme was explained and patients' relevant medical condition and current levels of PA were discussed. Patients were presented with information on the range of PA offered in their local area and they were given a tour of the leisure centre. They were provided with the opportunity to have a fitness appraisal, and then an individualised exercise plan was developed for the patient. During the remaining weeks of the programme, the patient was invited to engage in individually tailored and supervised exercise with a concessionary rate provided for gym attendance. This rate means that the entrance fee for accessing the leisure centre was discounted for those who were participating in the exercise on referral scheme. At 1 and 2 months, a brief (15-20 and 5 minutes, respectively) contact, by telephone or in person, was planned. At three months, the patient again had the opportunity to take part in an optional fitness appraisal and participate in a follow-up 30 minute consultation with the HFA. Therefore, overall the intervention aimed to have four formal contacts.

The SDT-grounded intervention pulled from guidelines for conducting exercise consultations [56] and strategies endemic to motivational interviewing [42]; in particular, listening skills, reflecting, handling resistance, and the autonomy supportive protocol developed for health counselors by Williams and colleagues [57]. The initial consultation covered a range of topics including a discussion of the benefits and risks of increased PA that was individualised to the client's perspectives. Patients were queried regarding the integration of an active lifestyle with their life values and their perceptions concerning their own barriers to and resources for change. Previous barriers to regular PA were discussed and the patient and HFA together engaged in implementation planning regarding increasing PA levels in the forthcoming week. The option for a fitness appraisal was also presented. Throughout the 3 month program, the HFAs in the intervention arm were requested to support successful PA engagement attempts, normalise failure, and facilitate the recalibration of implementation plans when necessary. During a final "booster" consultation (in which the fitness appraisal could be repeated if desired), focus was placed on the feelings the patient experienced during PA; HFAs were encouraged to recognise and support the internalisation of the patient's PA involvement. Working together, the aim was to develop a plan for post-scheme maintenance of PA.

The study was powered in terms of detecting between-arm differences in self reported PA in close to 500 participants, but unexpected scheduling demands facing the HFAs (e.g., needing to be away due to courses) meant that 347 patients were recruited in the trial and completed baseline assessments. Follow up rates at 3 and 6 months were 70% and 55% respectively. The participants in this trial were predominantly female (72.9%), middle-aged (*M*_Age _= 49.3 ± 13.6 years), and overweight or obese (90.3%) which corresponds to the demographics of the population typically participating in the exercise on referral scheme in the city in question and is similar to the sample from the PAC trial.

There were no significant differences in perceptions of HFA autonomy support among patients in the intensive or brief arm at 3 or 6 months, with values tending to be very high (6 and above on a 7 point scale) in each case. Physical activity levels and indicators of mental health and quality of life improved via participation in the scheme, regardless of whether the patients were in the intervention or control arm. These improvements were largely sustained at 6 months. Considering that this trial adopted a cluster design, between arm differences at follow up were analysed via multilevel modelling. There were no significant differences between the standard provision exercise on referral and the SDT-arm for self-reported PA, subjective vitality and the majority of the Dartmouth domains. However, at 6 months, participants in the intervention arm exhibited lower anxiety and depression scores (albeit the latter only approached significance; p < .07) and also rated their overall health (as assessed via the Dartmouth COOP Charts) to be significantly more positive.

Based on the observed between-groups differences, it could be questioned whether HFA autonomy support is relevant to patients' motivation and ensuing levels of moderate-vigorous PA. Grounded in SDT, a process model was tested to examine the social environmental and motivation-related processes predicting patients' mental health and PA intentions (at 3 months) and PA engagement at 6 month follow-up [58]. All of the participants were included in the analysis, regardless of whether they interacted with the HFAs from the standard provision or intervention arms. The patients' degree of self determination when commencing the program positively predicted their need satisfaction when exiting the scheme. Consistent with SDT [2], the degree to which the HFA (whether specifically trained in SDT-based principles and strategies or not) was autonomy supportive during the 3 month program also was positively linked to participants feeling more competent, autonomous, and related when the program terminated. Also consonant with SDT [2], if the patients reported experiencing higher need satisfaction at 3 months they also felt more self-determined at the conclusion of the scheme, with the latter variable corresponding to diminished depressive symptoms. There was also a direct link from need satisfaction experienced in the exercise on referral program and intentions to continue with one's PA engagement post-program. These intentions were significantly linked to PA levels assessed at follow-up.

In sum and consonant with SDT, the findings from the Empower trial indicate that autonomy support from PA advisors, greater autonomous motivation, as well as need satisfaction are relevant to the degree to which an exercise on referral program contributes to PA levels of patients after the program has concluded. Patients who demonstrated greater internalisation of their reasons for engaging in PA over the course of the 3-month scheme also exhibited more positive mental health (as reflected in decreased depression scores). These patterns of relationships were not dependent on whether the PA advisor was trained in SDT. With respect to the testing of theoretical tenets within this RCT, when contrasted with the standard provision service, there was no effect of the intervention on participants' perceptions of the autonomy support provided by their HFA and a number of the targeted outcomes at 6 months. Being exposed to HFAs who had received training in SDT principles and strategies did translate, however, into participants reporting more optimal physical and psychological functioning. Logistical challenges within the scheme itself and other issues that faced the research team more than likely compromised the adequacy of the HFA training and, indeed, implementation fidelity regarding components of the intervention. Overall, the results of this trial are in line with SDT predictions.

## The PESO (Promotion of Health and Exercise in Obesity) Trial

The PESO trial was initially conceived in an effort to provide experimental support to post-hoc findings from a previous weight loss trial showing that intrinsic motivation for participating in exercise predicted long-term weight control in initially overweight women [59]. In the obesity literature, PA is a consistent predictor of success in weight control [60,61]. However, motivation-related correlates of PA and exercise behaviors in this population are not well described. Thus, an underlying hypothesis of the PESO trial was that motivational aspects of PA engagement, namely those related to qualitative aspects of motivation, would play a key role in the ability to lose and maintain weight. Beyond the expected meditational role of exercise motivation in the effects of the intervention on exercise/PA behavior, it was also hypothesized that autonomous motivation could also benefit other weight-related behaviors, namely diet, with compounding effects on weight control [62,63].

Given that details of the study's design and protocol are described elsewhere [7,8], they are only briefly summarized here. The PESO study was a randomized controlled trial for overweight and mildly obese women (n = 239) consisting of a 1-year behavior change intervention and a 2-year follow-up period with no intervention. The intervention group attended 30 weekly/bi-weekly group sessions for approximately one year. The control group received a general health education curriculum, of similar total contact time, based on several educational courses on various topics (e.g. stress management, self-care). Measurement of key SDT variables - conducted at baseline, 4 months, and years 1, 2, and 3 - included the HCCQ [45], the Treatment and Exercise Self-Regulation Questionnaires [64,65], the Exercise Motivations Inventory 2 [66], the Exercise Locus of Causality scale [67], and the Exercise Intrinsic Motivation Inventory [68].

Intervention principles and the style of the intervention were based on SDT, with a special focus on increasing competence and autonomous motivation towards exercise and weight control. Intervention staff received training in the form of workshops and formal and informal training meetings, conducted by in-house as well as external experts in MI and SDT [37]. To create an autonomy-supportive environment, the intervention team's main goal was to promote participants' sense of ownership and internal locus of causality over their own behavior. This included encouraging choice and self-initiation (prescriptions, pressure, demands, and extrinsic rewards were mostly absent), providing a menu of options for behavior change (e.g. different types of PA), and encouraging congruence between values and life goals, and participants' lifestyles. Generally, the approach was to provide options and let people make their own decisions regarding PA, encouraging participants to find the activities they enjoyed the most and that they were most likely to retain for the future.

Drawing from the SDT literature, another environmental dimension relevant to motivation and need satisfaction is the provision of structure (37). In the PESO trial, issues related to safety and skills, setting and managing PA goals, monitoring PA, and dealing with personal barriers were covered, along with positive feedback of an informational nature. A 10-week dance curriculum was also available to prompt fun and enjoyment during activity, increase physical self-esteem and positive body image, and experiment with new activities. Overall, the intervention encouraged self-initiation and independent problem-solving, helping individuals explore their own motivations for treatment and define their personal treatment goals, while limiting external contingencies and controls [69].

Results showed that the program was highly successful in changing moderate and vigorous (M = +138 ± 26 min/week vs. controls, p < 0.001) and lifestyle PA (ES = 1.14 vs. controls, p < 0.001) at intervention's end (1 year). Furthermore, these differences remained highly significant at the year 2 follow-up [70]. The intervention also induced statistically significant changes in perceived need-support (ES = 1.01), treatment and exercise autonomous self-regulation (ES > 1.05), and also perceived competence (ES = 0.52), locus of causality (ES = 0.68), and enjoyment of exercise and PA (ES = 0.60), versus the control group [8]. Importantly, mediation analysis indicated that the motivational sequence proposed by SDT (see Figure 1) was empirically supported. Perceived need-support mediated the effects of the intervention on competence and autonomy need satisfaction in the exercise domain, which in turn predicted more autonomous forms of motivation. The role of the intervention, mediated by perceived autonomy and competence support, was particularly effective in increasing intrinsic motivation, which significantly predicted minutes of moderate and vigorous PA at year 2 [70]. In turn, PA at year 2 mediated the effects of the behavioral regulations on weight control at year 3 [27]. Interestingly, autonomous exercise motivation was also significantly *directly *associated with long-term weight loss (i.e., not mediated by PA), which could be due to spill-over effects on eating self-regulation [71,72].

The PESO trial provided clear results in support of using SDT to promote PA and long-term weight control. Still some issues pertaining to the implementation and methodology of this trial, along with those of the PAC and Empower trials are worthy of attention as they may inform future research efforts. These issues are discussed in the integrative section that follows.

## Summary, Limitations, and Considerations for Future Work

Although the trials were conducted in three different countries, with their inherent surface-level cultural disparities, varied in length and intensity, and entailed common and unique intervention components, they all provided substantial support for the hypothesized SDT motivational process model. Indeed, they were the first RCTs to test some or all of the sequential steps put forth by SDT in the PA promotion context (see Figure 1). These findings are aligned with the arguments of Deci and Ryan and existing evidence [73,74] suggesting that the psychological needs are universal and that the assumed social environmental predictors of more autonomous motivation and optimal functioning apply across nations.

Further, all three trials showed intervention effects (albeit some more comprehensively and robustly than others). In the PAC trial, significant between arm differences in quantity of motivation, quality (autonomous) motivation, and reported PA were exhibited at 6 weeks (mid intervention) as well as 13 weeks (end intervention) and quantity motivation effects were sustained in the post intervention phase. Moreover, intensive intervention arm participants showed greater decreases in body composition from 13 weeks to 25 weeks. In Empower, participants in the intervention arm exhibited lower anxiety scores and also rated their overall health to be significantly more positive at follow-up (6 months). Finally, with respect to the 1-year long PESO intervention, significant differences emerged between arms in perceived need support, perceived competence, autonomous motivation, locus of causality and enjoyment as well as PA at one year and follow-up (two years).

In terms of intervention content, this trio of interventions revealed many similarities with respect to their adoption of SDT principles to create autonomy-supportive contexts. For instance, as it is evident from Table 3, there are notable parallels between the ASSIST phase (5^th ^A) of the PAC trial, the individualized discussions within the Empower trial, and the group session of the PESO trial, particularly with respect to setting PA-related goals, aligning attempts to become more active with life goals, problem solving, and encouraging enjoyment of physical activity.

It is possible that some differences in the three programs might have led to differences in outcomes across the three trials. For instance, it is possible that contact with the HFAs in the Empower trial might have been too infrequent or short, or that group sessions in the PESO trial might have fostered more group support and thus met participants' relatedness needs to a higher extent than in the other trials.

Collectively then, the three particular trials indicate that to the extent that interventions influence psychological needs (through need-supporting environments), more autonomous motivation ensues, which in turn predicts positive PA and/or psychological outcomes. Moreover, these trials suggest that even when interventions are not entirely successful in affecting theory-based constructs (e.g., perceived autonomy support, perceived competence, autonomous self-regulation) versus a control or comparison condition, the same constructs can be found to predict positive behavioral and psychological outcomes in both conditions. Although the level of supportive evidence for SDT is lower in such cases, the theoretically-assumed model can be found to be operative even in the absence of a statistically significant "SDT-based intervention" effect. This is an important aspect bearing in mind the unpredictable and at times inherently need-supportive environments that can be found in many real world scenarios (e.g., fitness counseling, doctors' offices).

In addition, when considering the findings emanating from the three trials, it should be acknowledged that need satisfaction does not automatically ensue from supporting contexts. Rather, need satisfaction is assumed to result through a dialectic relationship between social contexts and individual characteristics [75]. Such findings also speak to the fact that comparative effects between contexts may be weakened by difficulties in creating non-need-supportive control conditions, as advisors/counselors most likely inherently have their participants'/patients' best interest in mind (i.e. support patients' needs). The findings from the process model tested in the Empower trial are consistent with this premise [58]. It would be ill-advised to purposefully create non-need supportive environments solely for the purposes of enhancing internal validity within the RCT design. From this, it is suggested that future research in this area would do well to use standard care as the control group condition, as in many medical-health RCTs, although this might lead to smaller intervention effects due to naturally occurring need support in these contexts. Moreover, future research could determine which techniques need to be delivered in these settings (above naturally occurring need support) to boost need satisfaction.

It is fair to say that the three trials reviewed have their share of limitations, to which we can add certain inevitable challenges that PA researchers face when conducting RCTs in the field. Notably, measurement tools and their implications for related analyses is one of the areas presenting challenging issues. One such concern pertains to the use of instruments assessing exercise motivation in sedentary individuals (e.g., at baseline), with stems like "I exercise because...", such as the Exercise Self-Regulation Questionnaire. These assessments may be troublesome as many individuals have been sedentary most of their lives and may lack the knowledge to formulate adequate answers. To address this limitation, researchers in the PESO trial, for instance, opted to use unadjusted measures (at 4 and 12 months) for some psychosocial exercise variables, and not pre-post change scores, to examine intervention effects. The advantage of having an adequate (standard care) control group was evident in this regard. On the same note, Teixeira and colleagues [76] observed an interesting phenomenon with exercise self-efficacy, which significantly decreased in the control group during the 1-year program but did not change in the intervention group (with significant time-group effects) [76]. That is, it appears that self-efficacy, and perhaps other related SDT-based constructs, could be artificially inflated at baseline in all subjects, again possibly from a lack of experiential knowledge regarding PA or from an initial social desirability effect. This was also found in the PAC trial with autonomous motivation [4]. This possibility warrants caution in interpreting pre-post change scores of these mediating variables.

Related to the above point, the PAC and Empower trials, among others, may have been affected by ceiling effects on motivational and social environmental variables. That is, high baseline/pre-intervention scores on SDT constructs may have prevented the finding of significant intervention effects on these variables. As mentioned above, in terms of assessing the degree of autonomy support provided by the HFA in the Empower trial [5], participants' scores on the HCCQ [45] were generally very high and not significantly different across the SDT intervention and standard provision arms. This finding also speaks to the point mentioned above with respect to limitations in achieving non-need supportive control groups.

Although the HCCQ was developed to assess the degree to which an advisor/behavioral consultant is autonomy supportive [36], this measure actually taps overall need satisfaction or environmental support. In this regard, it is not unreasonable to suspect that counselors or consultants who are involved in promoting behavioral change would naturally, without purposeful training, vary in the degree to which they are supportive of each of the needs that the HCCQ would appear to tap. Particularly after being exposed to a physical activity advisor (standard care or intervention) who is likely to be quite engaging, interested in the client/patient, enthusiastic about PA, and providing information on PA engagement, it is reasonable to expect that clients/patients might be prone to providing very positive overall ratings on the HCCQ. Such measurement issues point to the need to potentially develop more sensitive objective measures of the content and social environment manifested in PA promotion consultations [58]. Moreover, such issues might warrant that the fidelity of delivery of SDT-based interventions (and control conditions) be independently assessed in future studies.

In the psychology realm, there has been concern over "therapists drift", whereby clinicians, over time, drift away from firm adherence to their intervention training, implementing a previous or other approach [77]. This type of phenomenon may be equally relevant for those intervening in PA promotion and thus it would be important that interventionists' fidelity to SDT training protocol be regularly monitored and reported in the manner of some MI-based intervention studies (for example [78]). In the PAC trial, implementation was assessed in multiple manners including recording of sessions with the physical activity counselor and assessing for compliance to the IPAC protocol and to SDT [47]. In the PESO trial, although more could have been done to more strongly ensure protocol implementation, this was maximized by holding regular meetings to discuss fidelity issues and by the use of manipulation checks conducted by a senior interventionist during randomly assigned sessions [7,8]. In the Empower trial, the fidelity of intervention delivery was examined to some degree via observations of a sampling of one-on-one consultations within the SDT-based arm and meetings between members of the research team and the intervention arm HFAs [Rouse, Ntoumanis, Duda: The development of an observation assessment tool examining environmental support within physical activity consultation, submitted]. We suggest that future research in this area systematically include an implementation evaluation in addition to an outcome assessment. Indeed, Marcus et al. [79] argued that assessment of the fidelity of intervention delivery is one of the methodological issues that needs to be better addressed in future PA promotion RCTs.

In addition to matters of measurement, methodological concerns of a logistical nature also arise in conducting SDT-based PA promotion RCTs. One of these challenges pertains to the characteristics of intervention participants themselves. Specifically, the informed and voluntary participatory nature of the PAC, Empower, and PESO trials may have led to an overwhelming majority of participants with elevated initial levels of motivation, which could be largely autonomous, to make changes to their PA. It might be the case that SDT-based PA interventions differentially affect participants who voluntarily consent to being involved in a trial, namely in the sense that they are already more autonomous than non-participants or are becoming so. Moreover, they already have exercised their autonomy in agreeing to participate in the study in the first place. This complex issue may have compounded previously mentioned ceiling effects and potentially led to a weakening of the influence of these interventions. Although difficult to address, it is a limitation that future researchers should consider.

Another drawback relates to the specificity of participant samples in the aforementioned trials, which make it difficult to generalize the findings across populations. Indeed the typical participants in the three SDT-grounded trials just described were middle-aged overweight or obese women. Future studies should be conducted to evaluate intervention effects on groups that are more diverse in terms of their demographic (e.g., age, gender), motivational, and physical characteristics (e.g., body weight). Perhaps an international RCT promoting PA according to SDT propositions is in order. The three trials conducted to date would suggest that the health care context might be ideal for optimal reach and diversity of samples.

With respect to intervention procedures and to echo our point regarding delivery fidelity, SDT-based PA intervention studies thus far reflect a broader need to better report specific intervention techniques and their theoretical underpinnings [80]. Doing so would not only strengthen researchers' ability to make statements regarding an intervention's theoretical grounding but would also reinforce the external validity of these interventions and allow other researchers to draw on the strategies employed and replicate findings. Importantly, experts are now reflecting on the extent to which strategies used in MI [42] can be adopted in the future as the standard for what a typical SDT-based consultation centered intervention should look like, highlighting areas of agreement and areas where some discrepancies can be found (see Vanteenskiste, Williams, & Reniscow [81] this issue).

A related issue is the dearth of SDT-based studies that test intervention techniques and strategies independently to see how well each fairs in fostering the psychological needs and subsequently altering the quantity and/or quality of motivation and levels of PA (e.g, is one technique superior to another?). Undoubtedly, testing the combination of SDT-inspired techniques in the manner of the presented trials has been informative thus far. Yet, it is also by examining the unique effects of distinct techniques (such as the values interview) on mediators of PA changes that we can eliminate redundant components and optimize the most functional strategies. This should lead to more influential and more parsimonious interventions, which may be more cost-effective. Thus we suggest that researchers consider drawing from the "dismantling" approach and assign different groups of participants to receive either different, exclusive aspects of an intervention or combined components [82]. Such dismantling would help to pinpoint the most active ingredients in successful and less successful SDT interventions. Another approach would be to use sophisticated single-subject designs to sequentially test the different techniques in different orders and potentially in different PA promotion settings.

Current research in this area is also limited by failing to conduct economic evaluations of RCTs centered on PA promotion. In order to ascertain if such SDT-based interventions can be optimally implemented on a broader societal level, studies will need to determine whether these interventions (e.g., creating autonomy supportive contexts for behavioral change) are, or can be made, cost-effective (see Angus et al., [83] for an example of a cost-effectiveness evaluation from the PAC trial). Additionally, it should be noted that two of the trials presented involved relatively short-term intervention periods. For example, although the intervention in the Empower trial lasted 3 months and the intention was to have more theoretically informed contacts between HFA and the patients [6], logistical constraints meant that, in reality, there was an initial one hour consultation with many, but not all, of the patients also experiencing an end of the scheme 30 minute exit consultation. In PAC, intensive counseling patients received 6 sessions for an average of 206 minutes of counseling while in PESO participants received 30 sessions lasting 120 minutes each (approx. 3,600 minutes in total), albeit in relatively large groups of 25-30 women [7]. Given the intricacies of altering a complex behavior like PA [84] as well as the well-documented difficulties in maintaining initial success, future studies will need to consider the effectiveness, cost effectiveness, and feasibility of longer as well as more intense interventions. With more extended intervention programs, participants could be monitored regularly for lengthier time spans (e.g., 2 years or more) and, in some countries, over the course of different seasons. In such future work, it would be pertinent to consider the findings of Hillsdon et al.'s review [85] regarding the recommended frequency of intervention occasions. Interventionists should also take into account recent research on PA promotion consultations that point to the importance of including a post-program "top up" or follow up to facilitate participants' maintaining levels of PA engagement [86]. Studies that combine strategies focused on behavioral adoption *as well as *maintenance will provide further insight on the utility of SDT-based interventions in facilitating long-term changes in PA.

Any organized intervention effort toward behavior change in real life settings will hold its share of planning difficulties; attempts towards PA promotion are no exception. Such is the case when we consider the limited time that is available to train the interventionists who are charged with facilitating PA changes in a need-supportive manner. In the real-world, researchers, particularly those in primary care, have to deal with time constraints regarding the training of health care/PA promotion advisers. These advisors typically have limited backgrounds in psychology and models of behavioral change and also face have serious time constraints. In these cases, intervention effectiveness, which generally requires that behavior change programs be implemented in close to ideal conditions, is often sacrificed in hopes of greater efficiency and/or real-world applicability. Ameliorating these challenges may require experts to produce a 'roadmap' of more essential SDT strategies and concepts that should be conveyed in the training stages to those who will deliver these interventions (see [81], this issue). An alternative would be to teach autonomy supportive counseling in standard University courses, such as those for future Physical Activity Counselors, Registered Dieticians, or Medical Doctors.

Moreover, intervention researchers may wish to examine the threshold level of autonomous motivation that will optimize PA adoption and maintenance. Additionally, similar to what was done in the PAC trial [87], incorporating qualitative methodologies that draw on participants' experiences throughout the interventions will provide researchers with insight into how and when to optimize autonomous motivation. Finally, as alluded to in the opening section of this paper, progress in terms of the proper testing of mediation effects will need to be made, in order to obtain further support for SDT-assumed motivation sequences in developing and validating PA interventions. The PESO and PAC trials described herein represent important efforts in this direction. In closing, the authors wish to acknowledge the absence of any secondary analyses of trial findings presented in this review. As the number of SDT-based RCTs in the PA domain proliferates in coming years, experts are encouraged to generate a quantitative synthesis of the findings to guide future intervention efforts.

## Competing interests

The authors declare that they have no competing interests.

## Authors' contributions

MF and EG headed the structuring and writing of the manuscript as a whole and provided a first draft of several sections, including the section on PAC. JD took the lead on the writing of the Empower section and contributed to revising the manuscript. PT took charge of writing the PESO section and also contributed to editing the manuscript as a whole. All authors read and approved the final manuscript
